# An Extremely Difficult Case of Midwifery

**Published:** 1865-12

**Authors:** G. P. Paoli

**Affiliations:** Chicago


### AN EXTREMELY DIFFICULT CASE OF MIDWIFERY.

By G. P. PAOLI, M. D., Chicago.

*Presentation of the foot, arm, and umbilical cord before a very high standing segment of the head. Extraction with the forceps, of a living child. Adhesion of the placenta and extraction of the same.*

On the evening of the 14th November, I was called to see a poor Swedish woman, 29 years of age, well built, who had menstruated regularly from the age of 17, and previously had been delivered of three children. Her mother had been delivered of ten children, and always with great difficulty, and like her mother, this woman's previous delivery had been very tedious, for five years ago when she was delivered of her first child she suffered a three days protracted labor, and the physician who attended her had to extract, with instruments, a dead child. She suffered from that time of a rupture of the perineum, close to the intestinum rectum. The second birth she had a year and a half ago; after two days suffering she was delivered of a full-born child, with a breach presentation. Both of the above mentioned cases commenced with an early flow of liquor amnii.

The 13th of November, early in the morning, the patient experienced a sudden flow of liquor amnii, which continued to flow in small quantities. She called for a midwife of considerable experience, but feeling rather nervous about this case, the midwife requested my attendance. On examination I found the os uteri dilated to the size of a twenty-five cent piece. Liquor amnii had disappeared, and inside of uterus I felt a hand presenting; above this a larger round but mobile part of the fœtus, which I considered a part of the head. But after the lapse of a couple of hours, when I again examined her, I could not reach anything else but a small part of an extremity which was situated high over the right os pubis, and it was impossible to determine whether it was an arm or tibia; the rest of the pelvis seemed to be empty. The hand which I for a short time could feel in the pelvis, and the head being near the same, were facts which spoke in favor of a shoulder presentation, and the extremity which I could reach was a part of the arm; and my suspicion was still more strengthened on examination of her unequally dilated abdomen, where the broad uterus, bending to the right, seems to indicate an oblique situation of the fœtus. The palpitations of the heart of the fœtus were plainly heard below the umbilicus, about four inches in a transverse direction.

The greater part of the night she rested well; early in the morning she commenced to feel pains of a short duration. The 15th of November, at 9 o'clock, A. M., when I again examined her, the mouth of the uterus was very much contracted and swollen, that I could with difficulty introduce my finger. I was rather surprised to feel high up in the pelvis, on the right side, the toes, and back of this the outside margin of a foot, and behind this another foot, and this was all of the fœtus that I could discover. No pains appearing, the woman was quiet till 4 o'clock, P. M. On examination then I found plainly the head at the right side, and also a foot, while the margin of the other foot I could not without danger reach, and it seemed thus doubtful whether what I supposed on previous examinations to be the margin of a foot, was not the margin of a concealed hand.

A couple of hours later, regular pains appeared, and at 7 o'clock, when I repeated my examination, I found the uterus dilated to the size of a silver half dollar. Now I felt to the right of the foot a strong pulsating loop of the umbilical cord, and above this a part of a long small bone, which later proved to be the margin of ulna, and a little higher up a couple of joints of the fingers.

On the ground that the uterus was not sufficiently dilated, there was nothing to be done but to wait and observe the slowly increasing pains. During the middle of the night the pains commenced to be powerful and also very painful, and the uterus was dilated to the size of a silver dollar, and the foot and umbilical cord outside of the orificium, and the uterus in the upper part of the vagina. High up in the frontal part of the pelvis, I plainly felt an under arm laying across, which I could follow up to the elbow joint, and behind this above the entrance of the pelvis, I felt the head and small part of its suture.

Now the image of a complicated fœtus presentation began to be clear to my mind, because it was possible that the oblique irregular shaped uterus was the cause of the oblique direction of the fœtus. When we take into consideration the rather spacious entrance of the pelvis, and that to the left laying head, which for a time was prevented from entering into the same, while earlier and so much easier the right foot, arm, and umbilical cord had an opportunity to sink down in the right side of the pelvis. As neither the arm or foot could be brought back, and the threatening danger of the fœtus spoke for an early delivery. Under these circumstances there was scarcely anything to be done but turning by the feet, and extracting the fœtus, however doubtful the prognosis might seem, so far as regards the safety of the fœtus.

In the meantime the slow dilation of the uterus and undisturbed pulsations of the umbilical cord, allowed me to delay the delivery for a short time, which I rather chose, as I cherished a faint expectation that under the increasing pains the perceptible head, which I thought was occiput, would press down so much in the entrance that I could apply the forceps, and the expectation was fully realized. After I had patiently waited an hour, the slow pulsations of the umbilical cord indicated a quick operative delivery. In the meanwhile the uterus was well dilated, and the occipital part of the head was sunken further down the entrance. In my conviction that the prognosis was more favorable both for the woman and fœtus, I then tried the application of Professor Hodges forceps, and with great care and repeated slight rotations, I succeeded in applying the right branch in spite of the obstructions of the over arm and tibia.

The rest of the operation was not difficult, as the head and the under arm in front by a couple of tractions had been brought down beside the foot in the cavity of the pelvis; and by the next traction with the complete descent of the umbilical cord and arm, the head entered the outside parietes.

On examining the living child, I found the right hand and foot considerably swollen, and an impression on the right frontal bone, which testified the relative situation and pressure of the fœtus in the pelvis.

After the delivery, a sudden hemorrage appeared, which produced a very small feeble pulse with a sudden chilliness. I took such precaution as to master the hemorrage. After waiting a while for the contractions of the uterus, and subsequent expulsion of the placenta, I considered it would be judicious for the extraction of the same, as the hemorrage again appeared, and introducing my hand into the uterus, I discovered a partial adhesion of the same, and by careful manipulations, I extracted nearly the whole; the remaining part was expelled on the fifth day. On the 13th day this woman left her bed quite well.

